# Thinning Intensity Enhances Soil Multifunctionality and Microbial Residue Contributions to Organic Carbon Sequestration in Chinese Fir Plantations

**DOI:** 10.3390/plants14040579

**Published:** 2025-02-14

**Authors:** Ting He, Junjie Lei, Yuanying Peng, Ruihui Wang, Xiaoyong Chen, Zongxin Liu, Xiaoqian Gao, Peng Dang, Wende Yan

**Affiliations:** 1 College of Forestry, Central South University of Forestry and Technology, Changsha 410004, China; h1100097@163.com (T.H.); wang626389@163.com (R.W.); 20221200053@csuft.edu.cn (Z.L.); gxq19972022@163.com (X.G.); 2 National Engineering Laboratory for Applied Forest Ecological Technology in Southern China, Changsha 410004, China; lailxy1314@126.com; 3 College of Arts and Sciences, Saint Xavier University, Chicago, IL 60655, USA; pengyu@lewisu.edu; 4 College of Arts and Sciences, Governors State University, University Park, IL 60484, USA; xchen@govst.edu

**Keywords:** thinning, carbon sequestration, amino sugar, soil multifunctionality, phospholipid fatty acid, Chinese fir plantations

## Abstract

Soil multifunctionality is essential for the enhancement of soil carbon sequestration, but disturbances such as thinning practices can influence soil microbial activity and carbon cycling. Microbial residues, particularly microbial residue carbon (MRC), are important contributors to soil organic carbon (SOC), but the effects of thinning intensity on MRC accumulation remain poorly understood. This study evaluated the impact of four thinning treatments—control (CK, 0%), light-intensity thinning (LIT, 20%), medium-intensity thinning (MIT, 30%), and high-intensity thinning (HIT, 45%)—on soil multifunctionality in Chinese fir plantations five years after thinning. Soil nutrient provision, microbial biomass, enzyme activity, and microbial residue carbon were assessed. The results showed that thinning intensity significantly affected soil nutrient provision and microbial biomass, with MIT and HIT showing higher nutrient levels than CK and LIT. Specifically, MIT’s and HIT’s total nutrient provision increased by 0.04 and 0.15 compared to that of CK. Enzyme activity was highest in LIT (+0.89), followed by MIT (+0.07), with HIT showing a decline (−0.84). Microbial biomass, including bacterial PLFAs (B-PLFAs), fungal PLFAs (F-PLFAs), microbial biomass carbon (MBC), and nitrogen (MBN), was highest in CK and MIT, and lowest in HIT, with MIT showing a 0.13 increase compared to CK. Microbial residue carbon (MRC) accumulation was positively correlated with soil organic carbon (SOC), total nitrogen (TN), available nitrogen (AN), and easily oxidized organic carbon (EOC). The highest MRC content in the 0–20 cm soil layer was observed in MIT and CK (10.46 and 11.66 g/kg, respectively), while the MRC in LIT and HIT was significantly lower, reduced by 24% and 12%, respectively. These findings highlight the significant role of thinning intensity in microbial activity and carbon cycling. Medium-intensity thinning (MIT, 30%) was identified as the most effective approach for promoting microbial biomass and enhancing carbon cycling in Chinese fir forest soils, making it an optimal approach for forest management aimed at increasing soil carbon sequestration.

## 1. Introduction

Soil multifunctionality refers to the wide range of critical ecological functions that soil performs, and it has become a key indicator of soil quality [1,2]. Soil performs essential functions such as nutrient cycling, maintaining soil structure and stability, water filtration, microbial activity, and carbon sequestration [3]. It plays a critical role in recycling key elements like nitrogen, phosphorus, and carbon, while also providing structural integrity for plant roots and acting as a filter for water [4]. Soil also houses microorganisms that decompose organic matter, fix nitrogen, and contribute to carbon sequestration, thereby supporting climate change mitigation efforts [5]. Soil functions are vital for ecosystem health and productivity, supporting plant growth, regulating water flow, and recycling essential nutrients [3]. Soil maintains nutrient cycling, the structural integrity of plant roots, water filtration, and microbial activity, while acting as a carbon sink to help mitigate climate change [6]. It also plays a crucial role in carbon sequestration, supporting microorganisms that decompose organic matter, fix nitrogen, and enhance ecosystem sustainability [7].

Forest ecosystems are the largest terrestrial carbon pools, storing over 60% of the global carbon in terrestrial environments, with forest soils accounting for approximately 70% of total carbon sequestration [5,8]. These soils play a critical role in supporting multiple ecosystem functions, contributing significantly to the overall multifunctionality of forest ecosystems [9]. Soil microorganisms are integral to these processes as they help accumulate and transform soil organic matter (SOM) through their proliferation, metabolism, and decomposition [10]. Microbial residues, primarily composed of stable biomarker amino sugars, can persist in the soil long after microbial death, remaining stable for extended periods [11]. These microbial residues are crucial for soil organic carbon (SOC) sequestration, with some studies suggesting they contribute up to 80% of SOM and significantly influence SOC dynamics [12]. Factors such as soil nutrients [13], microbial biomass [14], and soil enzyme activity [15] affect microbial residue carbon (MRC) accumulation. The interaction between soil carbon (C) and nitrogen (N) is particularly important, as N can regulate plant photosynthesis, rhizosphere effects, and greenhouse gas emissions, thereby promoting SOC formation and the accumulation of MRC [16]. Soil enzymes and microbial biomass also play crucial roles in the formation, cycling, and decomposition of soil nutrients [17], impacting both the quantity and quality of SOC input and output. Recent studies have indicated that microbial residues account for approximately 51%, 47%, and 35% of the SOC in cropland, grassland, and forest ecosystems, respectively [18,19]. Additionally, forest rewilding [20], its succession stage [21], and its vegetation species [22] have been shown to affect the accumulation and stabilization of microbial residues, enhancing their contributions to its SOC. However, our understanding of how variations in microbial residue carbon accumulation affect SOC sequestration following thinning in forest plantations is still incomplete.

Thinning is a widely practiced silvicultural technique that enhances soil multifunctionality and microbial contributions to SOC sequestration [23]. It plays a crucial role in mitigating the negative effects of environmental stressors, such as precipitation changes, by maintaining microbial biomass and activity, thereby supporting soil health under shifting climatic conditions [24]. By adjusting thinning intensity, a forest’s structure and micro-environmental conditions are altered, which directly influences the composition, diversity, and functionality of its soil microbial communities [25]. These alterations in microbial communities affect microbial activity and nutrient cycling and contribute to SOC sequestration [26]. Research indicates that varying thinning intensities significantly influence soil microbial biomass, functional diversity, and activity, all of which play a crucial role in SOC sequestration [27]. Specifically, optimal thinning intensities enhance microbial functional diversity and activity, improving ecosystem functionality and carbon storage, while also promoting tree biomass growth, litter carbon storage, and understory plant diversity in managed forests [9]. Furthermore, optimal thinning intensities support microbial growth and activity, bolstering nutrient cycling and overall soil functionality [6]. Identifying the optimal thinning intensity is critical for the sustainable development of forest ecosystems. While many studies have focused on the effects of thinning on SOC stocks, the mechanisms underlying MRC accumulation and its role in carbon sequestration remain inadequately understood [28]. Clarifying the relationship between thinning practices, soil MRC, and carbon sequestration mechanisms is vital for enhancing forest management strategies and ensuring long-term ecosystem sustainability.

The Chinese fir (*Cunninghamia lanceolata*) is a fast-growing and important evergreen coniferous species native to China, widely regarded for its high timber quality and significant economic value [29]. As the dominant timber species in southern China, Chinese fir plantations (CFPs) span over 11 million hectares, making them one of the most extensively managed forest ecosystems globally [5]. These plantations play a critical role in timber production and carbon sequestration. However, Chinese fir plantations (CFPs) have recently become a focal point for the sustainable development of artificial forests, particularly concerning ecosystem sustainability and soil productivity [25]. To explore the effects of thinning on soil multifunctionality and microbial contributions to carbon sequestration, we implemented a control treatment and three thinning intensities in CFPs.

Our study aimed to assess the response of soil multifunctionality to varying thinning intensities and investigate the role of microbial residue carbon (MRC) in the soil carbon pool five years after thinning. We hypothesized that (1) soil nutrient provision and multifunctionality would peak under medium thinning intensity, and (2) the contribution of MRC to SOC would increase with thinning intensity. The objectives of this study were the following: (1) to investigate the effects of varying thinning intensities on soil multifunctionality, including nutrient provision, enzyme activity, microbial biomass, and overall ecosystem health in CFPs; (2) to examine the role of MRC in the soil carbon pool five years after thinning and assess how thinning intensity influences this process; (3) to determine the optimal thinning intensity for the enhancement of soil nutrient availability and multifunctionality, with a focus on identifying the threshold at which moderate thinning maximizes these soil properties; and (4) to evaluate how thinning intensity impacts the contribution of MRC to SOC sequestration and its implications for long-term carbon storage in forest ecosystems. This study aims to provide a scientific basis for sustainable forest management practices in CFPs by linking thinning practices with soil carbon dynamics and ecosystem functionality.

## 2. Materials and Methods

### 2.1. Study Site Description

This study was conducted at the Qingyang Lake State-owned Forest Farm, located in Ningxiang City, Hunan Province (28°10′–28°12′ N, 111°58′–112°05′ E) (Figure S1). The site is situated in the low hilly region of southern China, characterized by a typical continental monsoon humid climate. The average annual temperature is 16.2 °C, with total annual precipitation averaging 1438.8 mm, and the relative humidity is approximately 85%. The region experiences an annual frost-free period of 273 days, and the mean annual sunshine duration is 1737.6 h. The predominant parent rock in the study area is plate shale. The soil texture is loam, and the soil is mainly yellow soil, with a thickness of 40–100 cm. The Chinese fir plantations (CFPs) in the sample plot were established in 1998, and at the time of this study, the trees were 25 years old. In May 2018, the plantations underwent thinning treatments, which included a control (CK, 0%), light-intensity thinning (LIT, 20%), medium-intensity thinning (MIT, 30%), and high-intensity thinning (HIT, 45%). The thinning residues (such as branches, bark, and leaves) were left in place after the thinning treatment, remaining in their original state. The primary species in this area include *Cunninghamia lanceolata*, *Cinnamomum camphora*, *Pinus massoniana*, *Quercus glauca*, *Choerospondias axillaris*, etc. Additional details about the study site are provided in Table 1.

### 2.2. Experimental Design and Soil Sample Collection

The experiments were conducted in May 2023 using a split-plot design in the study area. The main factor was the four different thinning intensities selected for sampling in the Chinese fir plantations (CFPs), while the two soil layers (0–20 cm and 20–40 cm) were considered as sub-factors. A total of 12 plots were established, with three replications for each thinning intensity. Each plot measured 20 m × 20 m, and a minimum distance of 100 m was maintained between plots with different thinning intensities. For soil sampling, five soil cores were collected from each plot after removing the litter layer. Soil samples were taken from two depth intervals: the 0–20 cm and 20–40 cm layers. After the collection, visible animals, dead leaves, and stones were removed from the samples, which were then thoroughly mixed to form composite samples for each soil layer. A total of 32 soil samples were collected from the study site (4 thinning intensities × 2 soil depths × 4 replications).

The soil samples were placed in the incubator and promptly transported to the laboratory. A portion of the samples were sieved and stored at 4 °C in a refrigerator for the determination of soil enzyme activity and microbial biomass carbon and nitrogen (MBC and MBN). Another portion was frozen at −80 °C for an analysis of its phospholipid fatty acids (PLFAs) and amino sugars. The remaining naturally dry soil samples were sieved through 2 mm and 0.149 mm mesh sizes for the determination of the soil’s chemical properties.

### 2.3. Soil Chemical Property Analysis

Soil organic carbon (SOC) was determined using the K_2_Cr_2_O_7_-H_2_SO_4_ oxidation method. Total nitrogen (TN) was measured using the Kjeldahl method. Total phosphorus (TP) was determined by the molybdenum–antimony resistance colorimetric method. Available phosphorous (AP) was quantified using the HCl-H_2_SO_4_ extraction method. Available nitrogen (AN) was assessed using the alkaline (1 mol/L NaOH) diffusion method [30]. Ammonium nitrogen (NH_4_^+^) and nitrate nitrogen (NO_3_^−^) were measured with an Auto Analyzer 3 continuous flow analytical system (AA3, Brownluby, Hamburg, Germany) with KCl extraction. A TOC analyzer determined the dissolved organic carbon and nitrogen (DOC and DON) after K_2_SO_4_ extraction. Easily oxidized organic carbon (EOC) was quantified using the 333 mmol/L KMnO_4_ oxidation method. Soil microbial biomass carbon and nitrogen (MBC and MBN) were extracted by chloroform fumigation–K_2_SO_4_ and then determined using a TOC analyzer. The soil chemical properties are detailed in Table S1.

### 2.4. Soil Enzyme Activity Analysis

The activities of four soil hydrolytic enzymes involved in the C, N, and P cycles, namely β-Glucosidase (βG), 1,4-β-N-acetylglucosaminidase (NAG), Leucine aminopeptidase (LAP), and Acid phosphatase (ACP), were measured using a microplate fluorescence method with a multifunctional enzyme marker. The geometric mean of the hydrolase (GH) was calculated using Formula (1):

(1) $$\mathrm{GH}=\sqrt[{4}]{\beta G\times \mathrm{NAG}\times \mathrm{LAP}\times \mathrm{ACP}}$$

Enzyme activity was characterized by detecting fluorescence [31]. The procedure involved the following steps: first, 1 g of fresh soil was mixed with 125 mL acetate buffer (50 m mol/L) using a vortex oscillator. The resulting soil suspension was then used to measure the activity of the various enzymes. In the microtiter plate, the acetic acid buffer (50 m mol/L), the soil suspension (200 μL), the MUB or AMC standard material, and 50 μL of substrate (200 μmol/L) were added. The microtiter plates were incubated for 4 h at 20 °C in the dark, after which 10 μL NaOH (1 mol/L) was added into each hole. After 1 min, the reaction was determined, and fluorescence was measured using a multifunctional enzyme labeler (SynergyH4, Biotek, Winooski, VT, USA) with excitation at 360 nm and emission at 460 nm. The soil enzyme activities are listed in Table S2.

### 2.5. Soil Phospholipid Fatty Acid Analysis

Soil phospholipid fatty acids (PLFAs) were used as biomarkers to measure soil microbial living biomass [32]. The PLFAs were extracted from 3 g of freeze-dried soil using a chloroform–methanol–citrate buffer, then purified and methylated using a solid-phase extraction (SPE) column. The PLFAs were separated using Agilent 6850 GC (Agilent Technologies, Santa Clara, CA, USA) and quantified using MIDI (Microbial Identification System) software (Version 6.1, MIDI Inc., Newark, DE, USA), allowing for the extraction and identification of soil microbial groups [33].

### 2.6. Soil Ecosystem Multifunctionality Analysis

Soil ecosystem multifunctionality (SMF) was assessed in this study by categorizing all soil indices into three groups, namely nutrient provision (SOC, TN, TP, AN, AP, NH_4_^+^, NO_3_^−^, DON, DOC, and EOC), enzyme activity (βG, NAG, LAP, and ACP), and microbial biomass (MBC, MBN, bacterial PLFAs, and fungal PLFAs). These variables represent key soil properties related to matter stock and functionality, such as soil C sequestration, N storage, and fertility accumulation. For each group, a single function index was calculated, and an overall multifunctionality index was derived by averaging these values using the Tidyverse package in R 4.4.2 [34].

### 2.7. Soil Amino Sugar Analysis

Soil amino sugars, including Glucosamine (GlcN), Galactosamine (GalN), and Muramic acid (MurA) were determined by the pre-column derivatization of phthalaldehyde (OPA) and high-performance liquid chromatography (HPLC) [35]. GlcN and MurA served as markers for microbial residues, and their concentrations were used to calculate the C and N contents in amino sugars, as well as the microbial residue carbon contributions from fungi and bacteria. The calculation formulas for the fungal and bacterial microbial residue carbon (FRC and BRC) are as follows (Formulas (2)–(4)) [36]:

Fungal microbial residue carbon (FRC):

(2) $$\mathrm{FRC}=\left(\mathrm{GlcN}-1.16\times \mathrm{MurA}\right)\times 10.8$$

Bacterial microbial residue carbon (BRC):

(3) BRC=MurA×31.3

Microbial residue carbon (MRC):

(4) MRC=FRC+BRC

In these formulas, the units of FRC, BRC, and MRC are in g/kg. The molecular weights of GlcN and MurA are 179.17 and 251.23, respectively. The mean molar ratio for bacterial cells is 1.63, and the units for GlcN and MurA are in mmol/g. The conversion factor coefficients for bacteria and fungi are 31.3 and 10.8, respectively [36].

### 2.8. Statistical Analysis

A two-way analysis of variance (ANOVA) followed by least-significant difference (LSD) multiple comparison tests (at a significance level of *p* < 0.05) were performed to assess the effects of thinning intensity, soil depth, and their interactions on soil properties, enzyme activities, microbial biomass, and microbial residues. Correlation, regression, and redundancy (RDA) analyses were used to investigate the relationships between soil multifunctionality factors and microbial residues, identifying key factors influencing the soil microbial residues. A random forest model was used to predict the critical soil multifunctionality factors impacting MRC accumulation. Partial least squares path modeling (PLS-PM) was utilized to quantify the direct and indirect contribution of soil multifunctionality factors to MRC accumulation. All statistical analyses were performed using IBM SPSS Statistics 22, GraphPad Prism 8, and R 4.4.2 [34] software.

## 3. Results

### 3.1. Soil Ecosystem Multifunctionality

**Figure 1:**
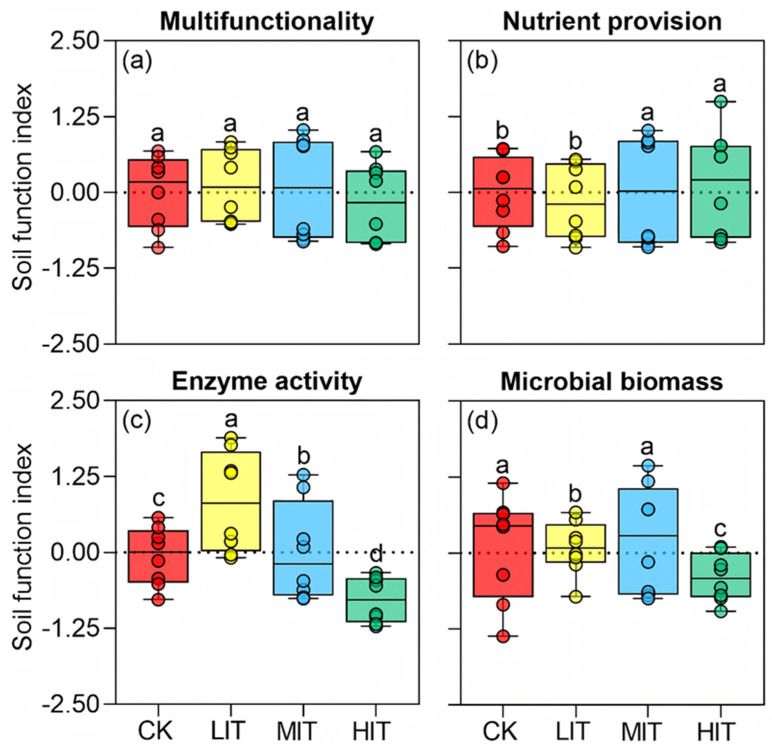
Variations in soil multifunctionality (**a**) and its nutrient provision (**b**), enzyme activity (**c**), and microbial biomass (**d**) under different thinning intensities in CFPs. Columns represent the mean ± standard error (n = 4). Different lowercase letters represent significant differences among thinning intensities of the CFPs (*p* < 0.05, Tukey’s test). Nutrient provision (**b**) includes SOC, TN, TP, AN, AP, NH_4_^+^, NO_3_^−^, DON, DOC, and EOC. Enzyme activity includes βG, NAG, LAP, and ACP. Microbial biomass includes MBC, MBN, B-PLFAs, and F-PLFAs. CK, control (0%); LIT, light-intensity thinning (20%); MIT, medium-intensity thinning (30%); HIT, high-intensity thinning (45%).

Figure 1 shows several key findings regarding the effects of different thinning intensities on the soil properties in Chinese fir plantations (CFPs). The thinning treatments did not significantly affect the overall soil multifunctionality index, including soil nutrient provision, enzyme activity, and microbial biomass across the different thinning intensities (control (CK), light-intensity thinning (LIT), medium-intensity thinning (MIT), and high-intensity thinning (HIT)) (Figure 1a). Overall nutrient provision, including SOC, TN, TP, AN, AP, NH_4_^+^, NO_3_^−^, DON, DOC, and EOC, showed significant differences among the thinning intensities (*p* < 0.05, Figure 1b, Table S1). The CK and LIT treatments exhibited lower nutrient provision values compared to MIT and HIT (Figure 1b), following the following trend: MIT = HIT > CK = LIT. Specifically, compared to CK, nutrient provision decreased by 0.16 in LIT, whereas it increased by 0.04 and 0.15 in MIT and HIT, respectively (Figure 1b). Enzyme activities, including βG, NAG, LAP, and ACP, varied significantly across the thinning intensities (*p* < 0.05, Figure 1c, Table S2). Compared to CK, enzyme activity increased by 0.89 in LIT and 0.07 in MIT but decreased by 0.84 in HIT. HIT exhibited the lowest enzyme activity, while CK had significantly lower activity than LIT and MIT (Figure 1c). Microbial biomass (MBC, MBN, B-PLFAs, and F-PLFAs) also varied significantly across the treatments (*p* < 0.05, Figure 1d). The highest values were observed in CK and MIT, with LIT showing slightly lower levels and HIT having the lowest microbial biomass (Figure 1d). Specifically, compared to CK, microbial biomass increased by 0.07 in LIT and 0.13 in MIT, but decreased by 0.51 in HIT (*p* < 0.05, Figure 1d).

This study (Figure 1) found that thinning intensity had no significant effect on the overall soil multifunctionality index, but significant differences were observed in nutrient provision, with medium- and high-intensity thinning (MIT and HIT) providing higher nutrient levels than the control (CK) and light-intensity thinning (LIT). Enzyme activities were lowest in HIT, and microbial biomass was highest in CK and MIT, but lowest in HIT.

### 3.2. Soil Phospholipid Fatty Acids (PLFAs)

**Figure 2:**
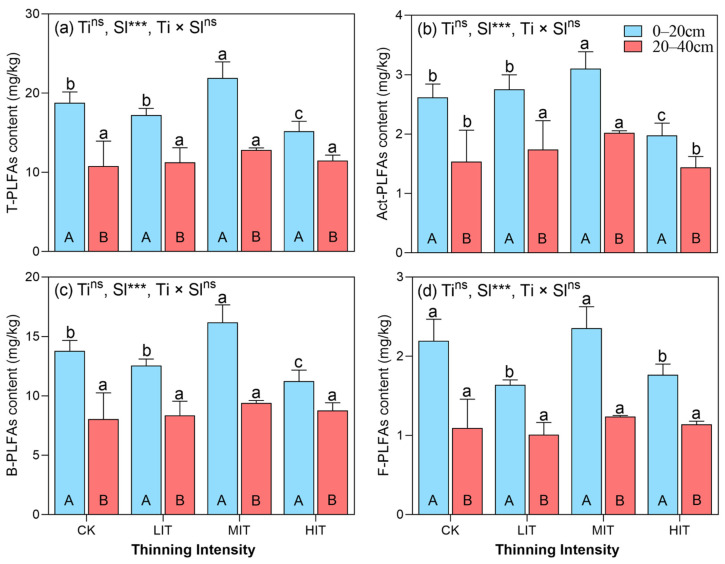
Vertical distribution of T-PLFA (**a**), Act-PLFA (**b**), B-PLFA (**c**), and F-PLFA (**d**) contents under different thinning intensities in CFPs. Columns represent the mean ± standard error (n = 4). Different uppercase and lowercase letters represent significant differences among the soil layers and thinning intensities of CFPs, respectively (*p* < 0.05, Tukey’s test). *, **, and *** indicate significance at *p* < 0.05, *p* < 0.01, and *p* < 0.001, respectively, and “ns” represents no significant differences (*p* > 0.05). CK, control (0%); LIT, light-intensity thinning (20%); MIT, medium-intensity thinning (30%); HIT, high-intensity thinning (45%). PLFAs, phospholipid fatty acids; T, total; Act, actinomycetes; B, bacteria; F, fungi. Ti, thinning intensity; Sl, soil layer; Ti × Sl, interaction between thinning intensity and soil layer.

The vertical distribution of total microbial biomass, including T-PLFAs (total PLFAs), Act-PLFAs (actinomycete PLFAs), B-PLFAs (bacterial PLFAs), and F-PLFAs (fungal PLFAs), across various thinning intensities in the CFPs (Figure 2) showed higher microbial biomass in the surface soil layer (0–20 cm) compared to the deeper layer (20–40 cm) (Figure 2). Thinning intensity influenced microbial biomass, with MIT promoting it and HIT reducing it, though soil depth was the primary factor driving microbial community distribution.

In the surface layer (0–20 cm), T-PLFA content was highest under MIT, followed by CK, LIT, and HIT. MIT increased T-PLFA content by 3.11 mg/kg (17%), whereas HIT decreased it by 3.61 mg/kg (19%) compared to CK. In the lower layer (20–40 cm), T-PLFA content was consistently lower across all treatments, with no significant differences among the thinning intensities (*p* > 0.05, Figure 2a). For Act-PLFAs, MIT had the highest content (3.10 mg/kg) in the 0–20 cm layer, followed by LIT, CK, and HIT. The lower layer showed significant reductions in Act-PLFA content across all thinning treatments, with MIT and LIT having higher values than CK and HIT. LIT exhibited the highest combined Act-PLFA content (2.75 mg/kg), followed by MIT (2.56 mg/kg) (Figure 2b). B-PLFA content was highest in the 0–20 cm layer under MIT (16.19 mg/kg), followed by CK, LIT, and HIT. The lower layer had a similar content across treatments. LIT had the highest combined B-PLFA content, while MIT had the lowest (Figure 2c). F-PLFA content was highest in the surface layer under MIT (2.35 mg/kg), followed by CK, HIT, and LIT. The lower layer (20–40 cm) had a consistently lower F-PLFA content across all treatments. The overall combined F-PLFA content was highest under MIT (1.80 mg/kg), followed by CK, HIT, and LIT (Figure 2d).

This study (Figure 2) found that microbial biomass was higher in the surface soil layer (0–20 cm) than in the deeper layer (20–40 cm). Thinning intensity affected microbial biomass, with MIT increasing and HIT decreasing it. MIT showed the highest levels of T-PLFAs, Act-PLFAs, B-PLFAs, and F-PLFAs, while HIT had the lowest. Soil depth played a key role in microbial distribution.

### 3.3. The Distributions of Soil BRC, FRC, and MRC Contents and Their Contribution to SOC

**Figure 3:**
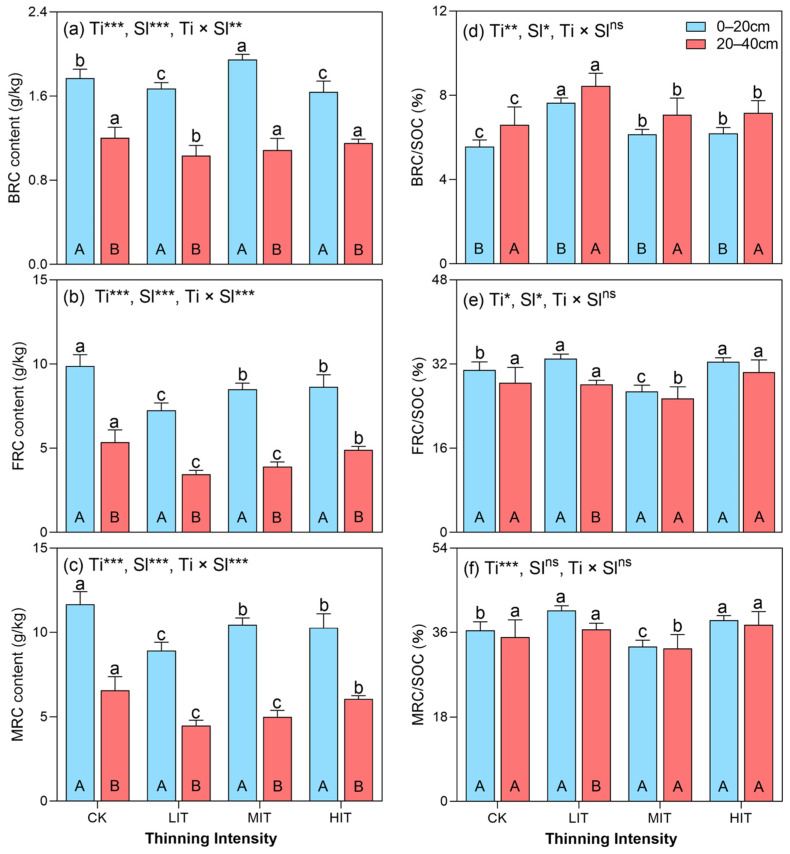
Vertical distributions of soil BRC (**a**), FRC (**b**), and MRC (**c**) contents and their contribution to SOC (**d**–**f**) under different thinning intensities in CFPs. Columns represent the mean ± standard error (n = 4). Different uppercase and lowercase letters represent significant differences among the soil layers and thinning intensities of the CFPs, respectively (*p* < 0.05, Tukey’s test). *, **, and *** indicate significance at *p* < 0.05, *p* < 0.01, and *p* < 0.001, respectively, and “ns” represents no significant differences (*p* > 0.05). BRC, bacterial residue carbon; FRC, fungal residue carbon; MRC, microbial residue carbon; SOC, soil organic carbon. CK, control (0%); LIT, light-intensity thinning (20%); MIT, medium-intensity thinning (30%); HIT, high-intensity thinning (45%). Ti, thinning intensity; Sl, soil layer; Ti × Sl, interaction between thinning intensity and soil layer.

The vertical distribution and contributions of BRC, FRC, and MRC contents were significantly influenced by thinning intensity, soil layer, and their interaction. Across both soil layers, microbial residue carbon (MRC), fungal residue carbon (FRC), and bacterial residue carbon (BRC) contents were higher in the 0–20 cm layer compared to the 20–40 cm layer, with significant variations among the thinning intensities (*p* < 0.001, Figure 3).

The BRC content showed the significant effects of thinning intensity (*p* < 0.001), soil layer (*p* < 0.001), and their interaction (*p* < 0.01). In the 0–20 cm layer, BRC content was highest under medium-intensity thinning (MIT, 1.95 ± 0.10 g/kg), followed by CK (1.77 ± 0.18 g/kg), LIT (1.67 ± 0.15 g/kg), and HIT (1.64 ± 0.09 g/kg). In this layer, MIT increased BRC content by 10% compared to CK, while LIT and HIT resulted in reductions of 6% and 8%, respectively. In the 20–40 cm layer, BRC content was highest under CK (1.20 ± 0.06 g/kg), followed by HIT (1.15 ± 0.19 g/kg), MIT (1.09 ± 0.17 g/kg), and LIT (1.03 ± 0.18 g/kg), with reductions of 4%, 9%, and 14%, respectively, under the thinning treatments compared to CK. The mean BRC content across both soil layers was significantly higher under MIT and CK than under LIT and HIT (Figure 3a).

FRC content was significantly influenced by thinning intensity (*p* < 0.001), soil layer (*p* < 0.001), and their interaction (*p* < 0.01). In the 0–20 cm layer, FRC content was highest under CK (9.89 ± 1.25 g/kg), followed by HIT (8.64 ± 0.63 g/kg), MIT (8.51 ± 0.76 g/kg), and LIT (7.25 ± 1.16 g/kg), representing decreases of 13–27% compared to CK. In the 20–40 cm layer, CK also had the highest FRC content (5.37 ± 0.34 g/kg), with reductions of 8–36% observed under the thinning treatments (HIT: 4.91 ± 0.48 g/kg; MIT: 3.91 ± 0.40 g/kg; LIT: 3.46 ± 1.25 g/kg). Across both soil layers, FRC content was significantly higher under CK, with MIT and HIT being comparable but higher than LIT (Figure 3b). MRC content was significantly influenced by thinning intensity (*p* < 0.001), soil layer (*p* < 0.001), and their interaction (*p* < 0.001). In the 0–20 cm layer, MRC content was highest under CK (11.66 ± 1.42 g/kg), with reductions of 24%, 10%, and 12% under LIT (8.92 ± 1.30 g/kg), MIT (10.46 ± 0.85 g/kg), and HIT (10.28 ± 0.70 g/kg), respectively. In the 20–40 cm layer, MRC content followed a similar pattern, with CK (6.57 ± 0.33 g/kg) showing the highest value, and reductions of 32%, 24%, and 8% observed under LIT (4.49 ± 1.41 g/kg), MIT (5.00 ± 0.55 g/kg), and HIT (6.06 ± 0.67 g/kg), respectively. Across both soil layers, CK had the highest mean MRC content, followed by HIT, MIT, and LIT (Figure 3c). The contributions of microbial residues to the SOC (BRC/SOC, FRC/SOC, and MRC/SOC) varied with the thinning intensity and soil layer. In the 0–20 cm layer, the BRC/SOC ratio was highest under LIT (7.65 ± 0.55%), followed by HIT (6.20 ± 0.41%), MIT (6.15 ± 0.40%), and CK (5.56 ± 0.47%), with a similar trend in the 20–40 cm layer. LIT increased BRC/SOC by 32% compared to CK (Figure 3d). The FRC/SOC ratio in the 0–20 cm layer was highest under LIT (33.03 ± 2.64%), followed by HIT (32.45 ± 2.07%), CK (30.89 ± 1.32%), and MIT (26.83 ± 1.53%). In the 20–40 cm layer, HIT showed the highest FRC/SOC ratio (30.50 ± 3.83%), with the value with MIT being significantly lower than in the other treatments (Figure 3e). For MRC/SOC, LIT had the highest ratio in the 0–20 cm layer (40.67 ± 3.16%), followed by HIT and CK, with MIT being the lowest. A similar trend was observed in the 20–40 cm layer. LIT and HIT increased MRC/SOC by 8% and 7%, respectively, compared to CK (Figure 3f). The proportion of microbial residue carbon within the SOC varied with the thinning intensity, significantly affecting the SOC levels across the soil layers. SOC was highest under CK, followed by MIT, HIT, and LIT, in the surface layer (0–20 cm), where LIT caused the greatest reduction (−32%), while MIT had little effect (−1%). In the lower layer (20–40 cm), SOC declined further, with reductions of 37%, 20%, and 16% under LIT, MIT, and HIT, respectively, though the differences among the thinning intensities were not statistically significant (*p* > 0.05, Table S1). On average, SOC was lowest under LIT (−34%), with smaller declines under HIT (−17%) and MIT (−9%). These findings show that light thinning had the most significant impact on SOC reduction, particularly in deeper soil layers, while moderate thinning had the smallest effect.

This study (Figure 3) found that thinning intensity affected the microbial residue carbon (BRC, FRC, and MRC) in Chinese fir plantations, with higher content in the 0–20 cm layer. Medium-intensity thinning (MIT) increased carbon, while light-intensity thinning (LIT) reduced it. BRC was highest under MIT, and FRC and MRC were highest in the control (CK). LIT caused the greatest reduction in soil organic carbon (SOC), especially in the deeper layer, while MIT had the smallest impact.

### 3.4. Correlation Between Microbial Residue Carbon and Soil Multifunctionality Factors

**Figure 4:**
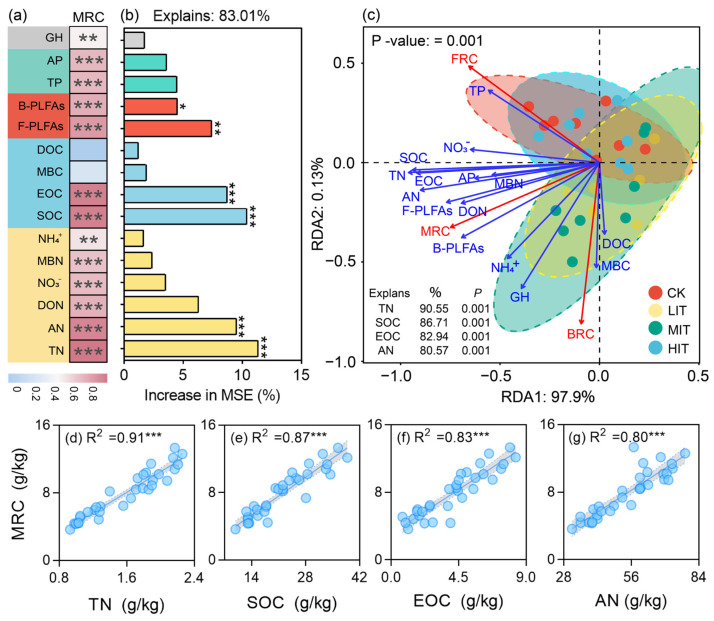
Correlation heatmap (**a**), random forest analysis (**b**), and redundancy analysis (**c**) of the relationship between soil biotic and abiotic factors and MRC under different thinning intensities in CFPs (n = 32). Regression analysis (**d**–**g**) of soil C, N fractions, and microbial PLFAs with MRC (n = 32). *, **, and *** indicate significance at *p* < 0.05, *p* < 0.01, and *p* < 0.001, respectively. SOC, soil organic carbon; TN, total nitrogen; TP, total phosphorus; AP, available phosphorous; AN, available nitrogen; MBN, microbial biomass nitrogen; MBC, microbial biomass carbon; EOC, easily oxidized organic carbon; NH_4_^+^, ammonium nitrogen; NO_3_^−^, nitrate nitrogen; DON, dissolved organic nitrogen; DOC, dissolved organic carbon; MRC, microbial residue carbon. PLFAs, phospholipid fatty acids; T, total; B, bacteria; F, fungi; Act, actinomycetes. GH, geometric mean of the hydrolase. CK, control (0%); LIT, light-intensity thinning (20%); MIT, medium-intensity thinning (30%); HIT, high-intensity thinning (45%).

Soil properties, especially TN, SOC, EOC, and AN, are key drivers of MRC variability, significantly influenced by thinning intensity. Microbial activity, indicated by B-PLFAs and F-PLFAs, plays a crucial role in MRC dynamics, highlighting the importance of microbial communities in carbon cycling under different thinning regimes in CFPs. MRC showed strong positive correlations with SOC, TN, AN, MBN, and EOC (*p* < 0.001), and moderate correlations with NH_4_⁺, NO_3_^−^, DON (*p* < 0.01), and GH (*p* < 0.05). B-PLFAs and F-PLFAs were also strongly correlated with MRC (*p* < 0.001, Figure 4a). Random forest analysis identified soil TN, AN, SOC, EOC, B-PLFAs, and F-PLFAs as the most critical factors explaining MRC variability (R^2^ = 0.83, *p* < 0.05, Figure 4b). RDA revealed the distinct clustering of samples based on their thinning intensities (CK, LIT, MIT, and HIT), with SOC, TN, EOC, and AN being the primary drivers of MRC distribution, accounting for 90.55%, 86.71%, 82.94%, and 80.57% of the variance, respectively (*p* = 0.001, Figure 4c). Although MBC, MBN, NH_4_⁺, NO_3_^−^, and DON also contributed significantly, the phosphorus fractions (TP and AP) showed weaker associations. Regression analyses showed strong linear relationships between the MRC and soil carbon (SOC, EOC) and nitrogen (TN, AN) fractions (*p* < 0.001, Figure 4d–g). Significant correlations between MRC and microbial PLFAs (B-PLFAs and F-PLFAs) further emphasize the role of microbial activity in MRC dynamics.

**Figure 5:**
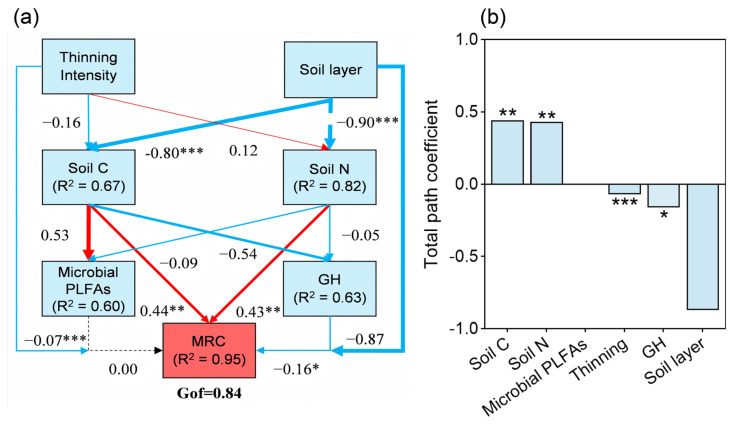
The partial least squares path model (PLS-PM) (**a**) and total path coefficient (**b**) showing the effects of thinning intensity, soil layer, soil C, soil N, microbial PLFAs, and GH on MRC accumulation in CFPs (n = 32). Red solid lines indicate positive paths, blue solid lines indicate negative paths, and black dashed lines indicate non-significant paths. The numbers beside the arrows represent path coefficients. *, **, and *** indicate significance at *p* < 0.05, *p* < 0.01, and *p* < 0.001, respectively. Soil C, soil carbon fractions (SOC, EOC); Soil N, soil nitrogen fractions (TN, AN); Microbial PLFAs, microbial phospholipid fatty acids (B-PLFAs, F-PLFAs); GH, geometric mean of four hydrolase enzymes (βG, NAG, LAP, and ACP); MRC, microbial residue carbon; Gof, goodness of fit.

Using the partial least squares path model (PLS-PM), we examined the relationship between soil multifunctionality factors and soil microbial residue carbon (MRC) accumulation, highlighting the relative contribution of thinning intensity and soil depth. This model shows that the goodness of fit value is 0.84. The soil carbon (SOC, EOC) and nitrogen (TN, AN) fractions had positive effects on MRC accumulation, with path coefficients of 0.44 and 0.43, respectively (*p* < 0.05, Figure 5a,b). In contrast, thinning intensity, soil enzyme activity (βG, NAG, LAP, and ACP), and soil layer negatively influenced MRC accumulation, with path coefficients of −0.07, −0.16, and −0.87, respectively (*p* < 0.05, Figure 5a,b). Soil microbial PLFAs (B-PLFAs, F-PLFAs) had no significant effect on MRC accumulation. The total path coefficients indicate that the soil carbon and nitrogen fractions are the key variables positively affecting MRC accumulation.

## 4. Discussion

### 4.1. Soil Multifunctionality Factors at Different Thinning Intensities

The results in Figure 1 highlight important insights regarding the impact of thinning intensity on soil multifunctionality in CFPs. The thinning treatments did not significantly influence the overall soil multifunctionality index, suggesting that soil multifunctionality is resilient to thinning interventions, consistent with findings from previous studies [2,4]. However, thinning significantly influenced key soil properties such as nutrient provision, enzyme activity, and microbial biomass.

Nutrient provision was significantly higher in the MIT and HIT treatments compared to CK and LIT. Specifically, nutrient provision decreased by 0.16 in LIT, while increasing by 0.04 and 0.15 in MIT and HIT, respectively, compared to CK. These findings align with studies reporting improved soil nutrient dynamics under moderate to intensive thinning [4]. Increased litter inputs and shifts in microbial activity following thinning are likely to enhance nutrient mineralization and availability, promoting nutrient cycling [37].

Enzyme activity varied significantly across the treatments, with the LIT treatment showing the highest enzyme activity, followed by MIT, while CK and HIT had lower activity. CK exhibited lower activity and HIT had the lowest. Compared to CK, enzyme activity increased by 0.89 in LIT and 0.07 in MIT, but decreased by 0.84 in HIT. This trend is consistent with Zhou et al. [38], who reported that thinning influences enzyme activities, involved in carbon and nitrogen cycling. The higher enzyme activity in LIT may result from a favorable environment for microbial processes, enhancing soil organic matter turnover and nutrient cycling [7]. In contrast, HIT likely reduced enzyme activity due to soil disturbance and organic matter depletion, disrupting microbial communities [3]. Interestingly, this non-monotonic response of enzyme activity may indicate a threshold effect, where too much disturbance (high-intensity thinning, HIT) leads to microbial community disruption, while medium-intensity thinning (MIT) allows for some microbial recovery [5,6]. Enzyme activity is a rapid indicator of microbial metabolism and nutrient cycling, offering valuable insights for short-term forest management decisions aimed at improving soil health. It reflects the current state of microbial processes, which can guide decisions to maintain soil vitality and ecosystem functioning in the immediate term [1,3].

Microbial biomass was also significantly influenced by thinning intensity. CK and MIT had a higher microbial biomass compared to LIT and HIT. Compared to CK, microbial biomass increased by 0.07 in LIT and 0.13 in MIT, but decreased by 0.51 in HIT. These results align with previous studies by Zhou et al. [38] and Wu et al. [39], which found that intense thinning disrupts soil microbial communities and reduces organic inputs. Microbial biomass, a key indicator of soil health, is closely linked to organic matter, which may be depleted under HIT, limiting microbial growth [4]. Medium-intensity thinning (MIT) appears optimal for maintaining microbial biomass and supporting soil health and ecosystem functions [40].

Microbial residues, such as amino sugars, act as long-term indicators of microbe-derived organic matter accumulation. These stable biomarkers provide insights into past microbial activity, offering a historical perspective on how silvicultural practices have influenced soil carbon storage over time. By monitoring amino sugars, we gain a deeper understanding of the cumulative effects of thinning on soil organic carbon (SOC) and soil health [12,23]. Both enzyme activity and amino sugars contribute to our understanding of how thinning influences habitat quality and biodiversity. While enzymes provide a snapshot of active microbial processes, amino sugars reflect the legacy of past microbial activity and its impact on long-term soil fertility. This distinction is crucial for making informed silvicultural decisions that optimize both short-term soil health and long-term ecosystem sustainability [2,6]. Amino sugar analysis is increasingly used to assess the impact of various agricultural practices, including tillage, fertilization, and organic amendments, on microbial residue accumulation [3,12]. This approach is valuable in evaluating the sustainability of Agri-Environment Schemes (AESs) by monitoring the microbial contributions to soil organic matter (SOM) under different land management strategies [2,6]. Furthermore, understanding how habitat and landscape gradients influence the formation of microbe-derived SOM can provide significant insights for sustainable land management practices across diverse ecosystems [3,6]. Soil nutrient dynamics exhibited a non-linear response to thinning intensity, with levels decreasing under LIT and increasing under MIT and HIT, except for indicators like available phosphorus (AP), dissolved organic carbon (DOC), and ammonium (NH_4_⁺) (Table S1). Under LIT, limited sunlight penetration may reduce understory vegetation growth, slowing litter decomposition and nutrient cycling. In contrast, MIT significantly improved nutrient levels, aligning with the Intermediate Disturbance Hypothesis (IDH) proposed by Connell [41], which suggests biodiversity peaks under moderate disturbance. Moderate thinning fosters optimal conditions for microbial activity, enhancing SOC formation and accumulation [42]. The non-monotonic development in nutrient levels across different thinning intensities suggests that there are thresholds at which the soil’s response shifts significantly, possibly due to changes in vegetation cover, microbial biomass, or soil structure. The mechanisms behind these shifts likely involve both biotic factors (e.g., microbial community composition, root exudation, and litter decomposition) and abiotic factors (e.g., soil moisture, temperature, and aeration) that are highly sensitive to thinning intensity [24,43]. Understanding these interactions is crucial for optimizing thinning practices that support both soil fertility and long-term carbon sequestration. These results highlight the importance of tailoring thinning practices to balance soil nutrient cycling, enzyme activity, and microbial biomass. While thinning does not alter the overall soil multifunctionality index, it significantly influences specific soil processes. Forest managers should consider these effects when designing thinning strategies to maintain soil health and promote forest sustainability [23].

Thinning intensity significantly influenced SOC levels across soil layers, with the greatest reduction observed under light-intensity thinning (LIT), particularly in the deeper soil layer. This decline may be attributed to reduced organic matter input from litter and root biomass, which are key contributors to SOC accumulation [37,44]. In contrast, medium-intensity thinning (MIT) had a minimal effect on SOC, suggesting that a balanced reduction in tree density can maintain soil carbon stability by preserving belowground carbon inputs [45]. The lower SOC content in the 20–40 cm layer regardless of thinning intensity may indicate the slower carbon turnover and reduced microbial activity in deeper soils [46]. Despite the SOC reductions under the thinning treatments, the lack of significant differences in the deeper layer (*p* > 0.05) suggests that long-term monitoring is needed to assess whether these effects persist over time. Further studies could explore the precise mechanisms behind these non-monotonic behaviors, particularly by monitoring the temporal changes in microbial and soil properties post-thinning to better understand the long-term impacts of thinning intensity on soil functioning.

### 4.2. Vertical Distributions of Phospholipid Fatty Acid (PLFA) Contents Under Different Thinning Intensities in CFPs

The vertical distribution of microbial biomass in the CFPs displayed clear stratification by soil depth, with a significantly higher biomass in the 0–20 cm layer compared to the 20–40 cm layer. This suggests that soil depth is the main factor influencing microbial distribution, with the surface layer supporting higher microbial activity due to greater resource availability. Thinning intensity also affected biomass, with MIT promoting and HIT reducing it, but its impact was secondary to soil depth. These results align with previous studies indicating that soil depth is a key determinant of microbial community distribution due to variations in organic matter, nutrient availability, and micro-environmental conditions [24,37].

MIT consistently resulted in the highest microbial biomass, followed by HIT, LIT, and CK. Moderate thinning improves microbial activity by enhancing resource availability, while excessive or minimal thinning may limit microbial growth due to resource scarcity or unfavorable conditions [47]. Interestingly, no significant interaction effects (between thinning intensity and soil depth) were detected, suggesting that thinning intensity and soil depth independently shape microbial community composition. This indicates that while thinning influences microbial biomass, it does not override the strong vertical gradients in soil properties [28].

The patterns of total PLFAs (T-PLFAs) and functional groups further emphasize the role of moderate thinning in enhancing microbial biomass in the upper soil layer. MIT likely creates an optimal balance between resource availability and microclimatic conditions, supporting diverse microbial communities [43]. In contrast, microbial biomass reductions in the 20–40 cm layer highlight the limited influence of thinning on deeper soil layers, where resource inputs are inherently lower [48]. These results support the ecological benefits of moderate thinning on soil microbial communities, promoting soil health and ecosystem resilience in managed forests like Chinese fir plantations [49]. Further research is needed to assess the long-term impacts of thinning on microbial diversity and its interactions with environmental factors, such as soil moisture and nutrient availability [50]. Overall, soil microbial communities in CFPs are predominantly controlled by soil depth, with thinning intensity playing a secondary role. MIT may be an effective management practice to enhance microbial biomass and ecosystem functioning, providing valuable insights for sustainable forest management. It is noteworthy that soil microbial activity may be associated with plot orientation selection. Previous studies have demonstrated that the slope aspect influences mean soil temperature, with south-facing slopes generally exhibiting higher levels of soil microbial activity [51]. Additionally, microbial activity in the soil is also influenced by moisture conditions [52].

### 4.3. Contribution of Soil MRC to SOC Accumulation and Its Driving Factors in Different Thinning Intensities

Higher residue contents were observed under MIT in the 0–20 cm layer, suggesting that MIT may optimize residue accumulation and enhance carbon storage, consistent with studies reporting increased surface soil carbon following thinning treatments [53]. Zhang et al. [43] found a 35–45% increase in surface soil carbon, which aligns with our results under MIT. The results indicate that thinning intensity and soil layer significantly influence the content and distribution of BRC, FRC, and MRC. It affects soil carbon dynamics by altering the amount and composition of forest residues [46]. Residue contents in the 0–20 cm layer were highest under MIT, followed by HIT, LIT, and CK, with all residue types (BRC, FRC, and MRC) showing this trend. This indicates that MIT promotes the accumulation of forest residues in the topsoil, critical for carbon sequestration. These findings align with Zhang X et al. [3], who observed that thinning can enhance soil carbon stocks by influencing surface litter and root biomass. Thinning increases organic material availability, enhancing nutrient cycling and microbial activity [44]. Gong et al. [47] observed a 30–50% increase in microbial residue carbon under high-intensity thinning, which matches our findings for MIT and HIT. This higher residue content under thinning can also be attributed to increased light penetration, which promotes understory vegetation growth and contributes to organic matter accumulation [54]. However, residue content was lower in the 20–40 cm layer, likely due to slower decomposition and reduced organic inputs at greater depths, in line with Zhou et al. [5], who found decreasing microbial activity and residue decomposition with soil depth. Thinning increases species diversity and understory vegetation, leading to an increase in decomposable litter. This, in turn, contributes to higher soil nutrient levels and enhanced microbial residue contributions to the organic carbon pool [55]. The quantity of organic matter added to the soil is proportional to thinning intensity, with higher intensity thinning resulting in more residue being left on the soil surface. The higher ratios of BRC/SOC, FRC/SOC, and MRC/SOC in the 0–20 cm layer under MIT and HIT suggest that thinning enhances the contribution to SOC, especially in the surface layers. Our study found the BRC/SOC ratio highest under LIT, followed by HIT and MIT (7.65%, 6.2%, and 6.15%, respectively). This supports the findings of Zhou et al. [5], who reported a 15–20% increase in the BRC/SOC ratio due to thinning. Thinning alters residue dynamics, promoting microbial activity and influencing soil carbon cycling [44]. In our study, MRC was highest under MIT in the 0–20 cm layer, with a 40% increase over CK, consistent with Xu et al. [24], who observed a 30–40% increase in microbial residue carbon following thinning. The observed interaction between thinning intensity and soil layer, particularly in the 0–20 cm layer, reflects the critical role of surface soil in carbon cycling. Shallow soil layers typically exhibit higher microbial activity and faster decomposition rates [39], explaining the higher residue contents and carbon contributions in these layers. Mazza et al. [48] also demonstrated a 20% increase in microbial activity in the surface layers following thinning, supporting the need for careful thinning management.

MIT significantly impacted biomass and microbial residues, which contributed to the SOC levels. The findings highlight the importance of careful thinning management to balance forest health and carbon sequestration, as well as microbial activity [56]. Microbial residues contribute 30–80% to SOC, depending on the soil type and SOC composition [18,57]. Our study found that MRC contributed 35–50% of the SOC in the 0–20 cm layer under MIT and HIT, aligning with these ranges. Thinning significantly increased the contribution of MRC to SOC, changing both the source and content of the SOC, which is consistent with prior research [45]. These findings emphasize thinning’s role in enhancing the microbial contributions to SOC in the CFP ecosystem.

### 4.4. Correlation Relationships of Soil Biotic and Abiotic Factors with MRC Under Different Thinning Intensities of CFPs

The findings of this study highlight the crucial role of soil properties and microbial communities in driving microbial residue carbon (MRC) dynamics in CFPs, with significant implications for forest management practices, particularly thinning. The relationships between MRC and SOC, TN, AN, MBC, and MBN are consistent with previous studies, indicating that soil C and N contents strongly influence microbial activity and carbon cycling [3]. SOC, as a primary substrate for soil microorganisms, positively correlates with MRC, suggesting that higher carbon availability promotes microbial activity, and microbial residue accumulation [4]. The associations between MRC and microbial phospholipid fatty acids (PLFAs), both bacterial (B-PLFAs) and fungal (F-PLFAs), emphasize the role of soil microbial communities in MRC dynamics, consistent with studies on microbial biomass and community composition in forest ecosystems [38,39]. Thinning alters the availability of carbon and nitrogen, affecting microbial communities and MRC accumulation [7]. The strong correlations between MRC and microbial PLFAs suggest that microbial activity, particularly bacterial and fungal activity, is a key in the decomposition of organic matter and MRC formation. Random forest analysis revealed that TN, SOC, EOC, AN, and microbial PLFAs are the most significant factors explaining MRC variability, supporting the importance of N and C availability in microbial dynamics and MRC accumulation in forest soils [5]. Previous studies have shown that forest thinning can increase soil carbon stocks by modifying soil nutrient status [47], with our findings highlighting the same. A redundancy analysis (RDA) confirmed that thinning significantly affects soil properties and microbial dynamics, with TN, SOC, EOC, and AN emerging as key drivers of MRC distribution. The positive associations of microbial biomass (MBC and MBN) and nitrogen fractions (NH_4_⁺, NO_3_^−^, and DON) with MRC suggest that thinning affects nitrogen cycling, further influencing MRC accumulation [5,50]. Regression analyses revealed strong linear relationships between MRC and TN, SOC, EOC, and AN, indicating that thinning, by modifying C and N availability, can have lasting effects on microbial decomposition and MRC formation [58]. Microbial communities, as indicated by their correlations with B-PLFAs and F-PLFAs, are integral to the decomposition processes driving MRC dynamics, consistent with the findings from Zhou et al. [38] and Zhang et al. [48]. This study demonstrates that soil properties, especially TN, SOC, EOC, and AN, are central to MRC variability under different thinning intensities in CFPs. Microbial activity, driven by bacterial and fungal communities, plays a vital role in MRC dynamics, underscoring the importance of microbial processes in carbon cycling. These results highlight the need for forest management strategies that consider the impact of thinning on soil carbon and nitrogen cycles, as well as on microbial communities, to optimize forest ecosystem carbon sequestration and sustainability [4].

MRC was positively correlated with soil C, N, P, microbial PLFAs, and GH, except for MBC and DOC, suggesting that these variables may be more transient and less directly involved in MRC accumulation [59]. This aligns with previous studies emphasizing the role of organic matter and microbial residues in soil fertility and carbon sequestration. The soil carbon and nitrogen fractions (SOC, EOC, TN, and AN) play a critical role in MRC accumulation, with higher levels promoting conditions favorable for microbial residue formation, crucial for soil carbon storage [47]. In contrast, thinning intensity, soil enzyme activities, and soil depth negatively impacted MRC accumulation, possibly due to reduced microbial activity and disturbances in the soil ecosystem. This finding is consistent with research showing that thinning alters nitrogen and phosphorus cycling in forest soils [5]. The negative path coefficients for thinning intensity (−0.07), enzyme activities (−0.16), and soil depth (0.87) suggest that thinning effects on MRC are complex and potentially adverse, particularly in deeper soil layers where microbial activity tends to decline [3]. Although soil microbial PLFAs (B-PLFAs and F-PLFAs) did not have a direct effect on MRC accumulation, this suggests that microbial community composition or activity does not necessarily correlate with microbial residue carbon production. This observation suggests that other factors, such as microbial turnover rates or the specific types of microbial residues produced, may play a more critical role in MRC dynamics than the microbial community structure alone [38]. Our overall findings highlight the importance of soil carbon and nitrogen fractions in promoting MRC accumulation, offering insights for forest management and carbon sequestration strategies in CFPs.

While this study provides valuable insights into the impact of thinning on microbial contributions to soil organic matter, several limitations should be acknowledged. The five-year post-treatment period restricted our ability to assess long-term effects on microbial dynamics and soil health. Additionally, this study focused primarily on microbial activity, omitting factors like microbial turnover rates, metabolic activity, and community diversity, which would have provided a deeper understanding of the processes at play. The results are also specific to Chinese fir plantations, and further research is needed to determine their applicability to other forest ecosystems. The variability in the results may be influenced by plot orientation, as south-facing slopes (with light-intensity thinning) and north-facing slopes (with medium-intensity thinning) differ in temperature, moisture, and radiation, which impact microbial activity and soil organic carbon (SOC) levels. Moreover, the potential side effects of thinning, such as soil compaction and erosion, were not assessed but could have counterbalanced some of the benefits observed. Future studies should address these limitations by extending the time horizon, incorporating microbial turnover and community diversity metrics, and investigating the broader applicability of the findings across forest types and environmental conditions. A more comprehensive approach that accounts for slope orientation and other environmental variables would also help isolate the effects of thinning on microbial communities and SOC dynamics. Finally, although we employed robust statistical methods like random forest analysis and redundancy analysis (RDA), a more thorough discussion of their limitations and uncertainties is needed, and future research should explore alternative statistical approaches to improve the reliability of the conclusions drawn.

## 5. Conclusions

This study highlights the varying effects of thinning intensity on soil microbial composition and activity in Chinese fir plantations (CFPs) five years after thinning. While thinning did not significantly alter the overall soil multifunctionality index, medium-intensity thinning (MIT) resulted in the highest microbial biomass and nutrient provision, with nutrient provision increasing by 0.04 compared to a 0.16 decrease under light-intensity thinning (LIT). MIT appears to be the most effective thinning treatment for enhancing microbial activity and supporting soil health in CFPs. However, the five-year post-treatment period limits our ability to assess long-term trends, necessitating further research to evaluate the persistence of these effects over time.

Microbial residue carbon (MRC) accumulation was positively correlated with key soil nutrients (total nitrogen [TN], soil organic carbon [SOC], easily oxidizable carbon [EOC], and available nitrogen [AN]), with MIT increasing MRC to 10.46 g/kg in the 0–20 cm soil layer, similar to the control (CK, 11.66 g/kg). In contrast, LIT and high-intensity thinning (HIT) reduced MRC by 24% and 12%, respectively, suggesting that MIT enhances soil carbon sequestration by promoting microbial activity. However, plot orientation may have influenced these results, as variations in the slope aspect can alter the soil microclimate and microbial responses. Future studies should control for environmental factors, such as slope orientation, to better isolate the effects of thinning on microbial communities and soil organic carbon (SOC) dynamics.

The highest microbial biomass was observed in the 0–20 cm soil layer, highlighting the importance of managing soil depth in thinning practices aimed at maximizing carbon sequestration. Additionally, microbial phospholipid fatty acids (PLFAs), particularly bacterial (B-PLFAs) and fungal (F-PLFAs) markers, were strongly associated with MRC accumulation, emphasizing the role of microbial activity in soil carbon dynamics. However, this study focused primarily on microbial activity without considering microbial turnover rates, metabolic activity, or community diversity. These factors are essential for a deeper understanding of microbial contributions to soil processes. Future research should incorporate these aspects to provide a more comprehensive perspective on microbe-mediated soil carbon sequestration.

While the statistical tools used in this study, such as random forest analysis and redundancy analysis (RDA), were effective, their inherent limitations and uncertainties must be acknowledged. These methods assume simplified relationships that may not fully capture the complexity of microbe-soil interactions. Future studies should explore alternative statistical approaches or conduct more comprehensive sensitivity analyses to improve model accuracy and better quantify uncertainties.

Our findings suggest that MIT is the most effective thinning treatment for the enhancement of microbial biomass, nutrient cycling, and carbon sequestration in CFPs. This supports sustainable forest management and climate change mitigation through improved soil carbon storage.

## Figures and Tables

**Table 1:** The characteristics of the study site.

Thinning Intensity	Altitude (m)	Slope Aspect	Gradient (°)	Mean DBH (m)	Average Tree Height (m)	Reserved Density	Canopy Density
CK	204	W	25	17.4	14.3	1353 ± 11.0	0.9
	186	S	23	18	14.2		
	188	SW	24	18.3	14.5		
LIT	190	S	26	19.4	14.3	1082 ± 7.2	0.8
	209	SW	24	18.8	14.0		
	189	S	24	19.6	14.3		
MIT	193	NW	25	20.1	14.6	947 ± 5.7	0.75
	195	N	26	19.9	14.5		
	190	S	24	20.3	14.4		
HIT	231	SE	25	21.3	15.2	750 ± 6.2	0.68
	201	W	24	20.9	15.1		
	186	SW	24	20.2	14.8		

Note: CK: control (0%); LIT: light-intensity thinning (20%); MIT: medium-intensity thinning (30%); HIT: high-intensity thinning (45%); Mean DBH: mean diameter at breast height.

## Data Availability

The original contributions presented in this study are included in the article/Supplementary Material. Further inquiries can be directed to the corresponding author(s).

## Supplementary Materials

The following supporting information can be downloaded at: https://www.mdpi.com/article/10.3390/plants14040579/s1.
