# Supplementary material for: Thinning Intensity Enhances Soil Multifunctionality and Microbial Residue Contributions to Organic Carbon Sequestration in Chinese Fir Plantations
Source: Plants (Basel). 2025 Feb 14;14(4):579. doi: 10.3390/plants14040579 (PMC11858922; doi:10.3390/plants14040579)
Supplement: Supplementary file 1 [file plants-14-00579-s001.zip › plants-3451618-supplementary.pdf]

## Supplementary materials

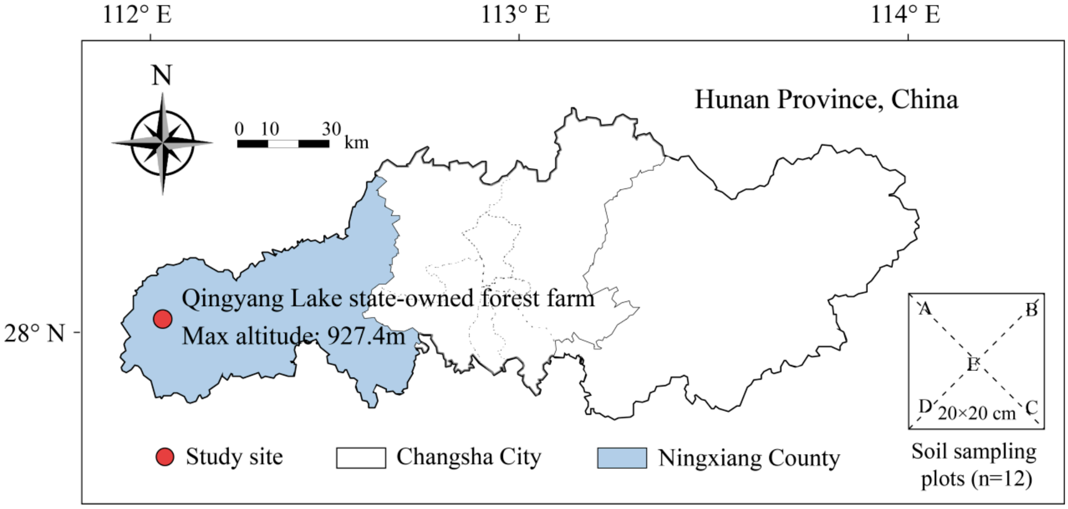


**Figure S1.** Study area of the Qingyang Lake State-owned Forest Farm in Ningxiang, Hunan, China.

**Table S1.** The soil chemical properties with different thinning intensities of CFP.

| Index | Soil layer | Thinning intensity | | | | Significance | | |
|---|---|---|---|---|---|---|---|---|
| | | CK | LIT | MIT | HIT | Ti | Sl | Ti×Sl |
| SOC (g/kg) | 0-20cm | 32.38±5.44Aa | 21.93±1.65Ab | 31.90±3.27Aa | 26.70±4.06Aab | ** | *** | ns |
| | 20-40cm | 19.49±6.01Ba | 12.29±1.45Ba | 15.53±1.82Ba | 16.36±2.18Ba | | | |
| TN (g/kg) | 0-20cm | 1.98±0.18Aa | 1.80±0.06Aa | 2.02±0.17Aa | 2.06±0.14Aa | * | *** | ns |
| | 20-40cm | 1.33±0.24Ba | 1.07±0.12Ba | 1.07±0.08Ba | 1.30±0.16Ba | | | |
| TP (g/kg) | 0-20cm | 0.23±0.02Ab | 0.20±0.01Ab | 0.22±0.01Ab | 0.34±0.04Aa | *** | *** | * |
| | 20-40cm | 0.20±0.02Aab | 0.16±0.02Bb | 0.17±0.01Bb | 0.23±0.04Ba | | | |
| AP (g/kg) | 0-20cm | 3.04±0.53Ac | 3.82±0.34Abc | 4.11±0.27Aab | 4.96±0.53Aa | ** | *** | * |
| | 20-40cm | 2.17±0.54Ba | 2.32±0.47Ba | 2.42±0.46Ba | 2.40±0.52Ba | | | |
| AN (mg/kg) | 0-20cm | 61.44±4.18Ab | 60.41±1.32Ab | 71.22±1.75Aa | 70.86±5.44Aa | ** | *** | * |
| | 20-40cm | 47.06±7.11Ba | 36.65±3.96Ba | 40.38±0.84Ba | 47.32±5.32Ba | | | |
| $NH_4^+$ (mg/kg) | 0-20cm | 7.81±1.80Aab | 7.85±2.10Aab | 9.50±3.32Aa | 3.62±1.54Ab | ** | *** | ns |
| | 20-40cm | 5.52±2.18Ba | 4.37±1.11Ba | 4.37±1.41Ba | 2.29±1.02Ba | | | |
| $NO_3^-$ (mg/kg) | 0-20cm | 3.64±0.72Ab | 4.50±1.02Aab | 3.90±0.62Aab | 5.73±0.98Aa | *** | *** | ns |
| | 20-40cm | 2.00±0.21Bb | 3.47±0.30Ba | 2.24±0.19Bb | 3.51±0.63Ba | | | |
| DON (mg/kg) | 0-20cm | 21.96±1.89Ac | 28.14±1.72Aab | 25.37±1.97Abc | 32.90±3.52Aa | *** | *** | ns |
| | 20-40cm | 16.63±3.23Bab | 19.02±0.95Bab | 14.06±2.72Bb | 20.32±1.14Ba | | | |
| MBN (mg/kg) | 0-20cm | 31.71±7.46Aa | 28.77±14.79Aa | 25.79±4.59Aa | 22.17±4.82Aa | ns | ** | ns |
| | 20-40cm | 24.37±6.04Aa | 11.93±4.45Aa | 20.33±9.40Aa | 13.82±4.10Aa | | | |

| | | | | | | | | |
|---|---|---|---|---|---|---|---|---|
| MBC（mg/kg） | 0-20cm | 198.17±87.78Aab | 236.33±38.19Bab | 281.50±72.49Aa | 115.83±3.75Ab | *** | * | *** |
| | 20-40cm | 117.50±15.74Ab | 370.50±27.21Aa | 77.83±23.02Bb | 106.00±3.89Bb | | | |
| DOC（mg/kg） | 0-20cm | 172.85±15.03Aab | 171.33±7.31Aab | 193.88±24.34Aa | 154.20±6.13Ab | *** | ns | ns |
| | 20-40cm | 171.68±17.33Aab | 194.48±23.65Aa | 178.08±12.11Aa | 137.03±9.68Bb | | | |
| EOC（g/kg） | 0-20cm | 6.95±1.18Aa | 4.68±0.62Ab | 6.94±0.52Aa | 5.01±0.88Aab | *** | *** | * |
| | 20-40cm | 4.23±0.38Ba | 1.09±0.27Bc | 2.16±0.40Bb | 2.24±0.36Bb | | | |

**Note:** All data are presented as the mean ± standard error (n = 4). Different uppercase and lowercase letters represent significant differences of soil chemical characteristics at different thinning intensities and soil layers of CFP at $p < 0.05$ level, respectively (Turkey's test). *, **, and *** indicate significance at $p < 0.05$, $p < 0.01$, and $p < 0.001$, respectively, and "ns" represents no significant differences ($p > 0.05$). CK, control (0%); LIT, light-intensity thinning (20%); MIT, middle-intensity thinning (30%); HIT, high-intensity thinning (45%). SOC, soil organic carbon; TN, total nitrogen; TP, total phosphorus; AP, available phosphorous; AN, available nitrogen; $NH_4^+$, ammonium-nitrogen; $NO_3^-$, nitrate-nitrogen; DON, dissolved organic nitrogen; MBN, microbial biomass nitrogen; MBC, microbial biomass carbon; DOC, dissolved organic carbon; EOC, easily oxidized organic carbon. Ti, Thinning intensity; Sl, Soil layer; Ti×Sl, Interaction between thinning intensity and soil layer; CFP, Chinese fir plantations.

**Table S2.** The soil enzyme activities with different thinning intensities of CFP.

| Index (nmol $h^{-1}g^{-1}$) | Soil layer | Thinning intensity | | | | Significance | | |
|---|---|---|---|---|---|---|---|---|
| | | CK | LIT | MIT | HIT | Ti | Sl | Ti×Sl |
| β G | 0-20cm | 13.26±2.39Ab | 47.00±3.64Aa | 21.83±9.80Aa | 11.32±4.29Ab | *** | *** | ** |
| | 20-40cm | 11.96±3.16Ab | 24.66±4.73Ba | 7.46±1.43Bb | 7.65±1.94Ab | | | |
| NAG | 0-20cm | 10.49±0.61Abc | 17.80±1.97Aa | 14.96±2.76Aab | 8.42±1.35Ac | *** | *** | *** |
| | 20-40cm | 5.91±0.18Ba | 5.68±1.26Ba | 5.18±1.98Ba | 4.11±0.36Ba | | | |
| LAP | 0-20cm | 30.19±8.67Ab | 60.64±8.93Aa | 45.27±7.63Aab | 28.76±4.05Ab | *** | *** | ns |
| | 20-40cm | 12.89±1.95Bb | 37.27±5.67Ba | 17.33±9.79Bb | 10.31±2.88Bb | | | |
| ACP | 0-20cm | 822.09±65.16Aa | 678.39±59.09Aab | 623.90±138.51Aab | 388.04±26.00Ab | *** | *** | ns |
| | 20-40cm | 606.47±122.36Ba | 591.50±42.84Aa | 519.73±73.98Aa | 297.20±23.71Bb | | | |
| GH | 0-20cm | 42.62±2.97Abc | 76.32±5.54Aa | 54.24±11.12Ab | 31.46±3.05Ac | *** | *** | ** |
| | 20-40cm | 27.01±3.32Bb | 41.42±3.09Ba | 22.68±3.18Bbc | 17.42±2.12Bc | | | |

**Note:** Four soil hydrolytic enzyme activities which involved in soil carbon, nitrogen, and phosphorus cycles in different thinning intensities of CFP. All data are presented as the mean ± standard error (n = 4). Different uppercase and lowercase letters represent significant differences of soil chemical characteristics at different thinning intensities and soil layers of CFP at $p < 0.05$ level, respectively (Turkey's test). *, **, and *** indicate significance at $p < 0.05$, $p < 0.01$, and $p < 0.001$, respectively, and "ns" represents no significant differences ($p > 0.05$). CK, control (0%); LIT, light-intensity thinning (20%); MIT, middle-intensity thinning (30%); HIT, high-intensity thinning (45%). βG, β-Glucosidase; NAG, 1,4-β-N-acetylglucosaminidase; LAP, leucine aminopeptidase; ACP, acid phosphatase; GH, the geometric mean of the hydrolase. Ti, Thinning intensity; Sl, Soil layer; Ti×Sl, Interaction between thinning intensity and soil layer; CFP, Chinese fir plantations.
